# Survival after heart transplantation for non-metastatic primary cardiac sarcoma

**DOI:** 10.1186/s13019-016-0540-x

**Published:** 2016-10-03

**Authors:** Hua Li, Shouguo Yang, Hao Chen, Zhaohua Yang, Tao Hong, Yingyong Hou, Chunsheng Wang

**Affiliations:** 1 Department of Cardiac Surgery, Zhongshan Hospital, Fudan University, No. 180 Fenglin Road, Shanghai, China 200032; 2 Department of Pathology, Zhongshan Hospital, Fudan University, Shanghai, China

**Keywords:** Sarcoma (heart), Cardiac tumors (primary), Transplantation, heart, Outcomes (survival), Adjuvant/neoadjuvant therapy

## Abstract

**Background:**

Heart transplantation is an uncommon treatment for unresectable and non-metastatic primary cardiac sarcomas, and the role of it is unclear. This study aims to offer a survival analysis of it.

**Methods:**

This study consists of 6 patients from our institution and 40 patients identified in a literature search who underwent heart transplantation for non-metastatic primary cardiac sarcomas. Seven patients with unresectable cardiac angiosarcoma who received palliative therapies at our institution were included for comparison. All the clinicopathologic data were collected, retrospectively reviewed and statistically analyzed.

**Results:**

Among the 46 patients receiving heart transplantation for primary cardiac sarcomas, the overall median survival was 16 months (2–112 months). The most common histologic type receiving heart transplantation was angiosarcoma. Its median survival time after heart transplantation (*n* = 14) was much less than that of other histologic types (*n* = 31) (9 vs 36 months; *P* = 0.002), which means it was not different from the median survival of 8 months for patients (*n* = 7) receiving palliative therapies (*P* = 0.768). The patients with grade 2 cardiac sarcomas (*n* = 5) survived much longer after heart transplantations than patients with grade 3 tumors (*n* = 15) (mean survival: 85 vs 18 months; *P* = 0.006). Neoadjuvant or adjuvant chemotherapy didn’t provide survival benefits after heart transplantation.

**Conclusions:**

Cardiac angiosarcoma seems to be not the proper indication of heart transplantation. The role of heart transplantation in other histologic subtypes still remains undefined. Lower grade and less aggressive histologic subtypes benefit more from heart transplantation.

**Electronic supplementary material:**

The online version of this article (doi:10.1186/s13019-016-0540-x) contains supplementary material, which is available to authorized users.

## Background

Primary cardiac sarcomas (PCS) are rare diseases. PCS usually carry a poor prognosis and surgery remains the most effective treatment [1]. Complete resection of localized PCS with microscopically negative margin (R0) greatly improves the prognosis [2, 3]. Microscopically positive margin (R1) resection or partial resection fails to provide benefits [2, 4]. Heart transplantation (HTx) appears to be the last surgical resort in the hope of R0 resection of tumors that are deemed unresectable with conventional surgical techniques. However, fear of exaggerated metastatic rate by immunosuppressant therapy and limited donor availability restricted the utilization of HTx. The role of HTx for PCS remains controversial. According to previous reports [5–27], HTx for PCS produced a broad range of survivals from only several months to almost a decade. Some reports denied the benefit of HTx [12, 23], whereas a few reports supported it [8, 16]. Actually, PCS are a heterogenous group of soft tissue sarcomas with a wide spectrum of clinical behaviors. Identifying the prognostic factor is important for selecting patients who stand a chance to benefit from HTx.

Unlike the field of liver transplantation for hepatocellular carcinoma, data for the HTx for PCS are sparse. Most of the studies presented in the literature were either single case reports or small case series. There was only one large series report published in the year 2000 which [16] included 21 cases of HTx for malignant cardiac tumors. As there is insufficient information about the survival analysis of HTx for PCS, this study is aimed to investigate this issue with all the data available. Combined data provide a better insight into this subject.

## Methods

### Study design, patient selection, and patient variables

This study protocol adheres to the principles of the Declaration of Helsinki and has been approved by the Medical Ethics Committee of Zhongshan Hospital Affiliated to Fudan University. It enrolled 13 patients from our institution (FO) and 40 patients from the literature (FL) [5–27]. A HTx group and a non-HTx group were created covering all the 53 patients.

The 13 patients FO were identified by a database searching conducted in our institution. Six of them underwent HTx for unresectable and non-metastatic PCS from year 2003 to 2014. The rest 7 patients presented an unresectable primary cardiac angiosarcoma (AS) with no distant metastasis at the same period. Due to the patient intention, limited affordability or limited donor availability, they received palliative treatment only.

The 40 patients FL were identified by a comprehensive search of PubMed conducted on Mar 1, 2016 for articles about patients who underwent HTx for PCS. Search terms of “((primary cardiac tumor) OR primary cardiac sarcoma) AND heart transplantation” were used for questing with no limitation of publication date and 391 articles in total(published from 1972 to 2016) were gathered. Among them, 368 articles were excluded as they did not present cases of HTx for PCS. A thorough review of the remaining 23 articles [5–27] (published from 1989 to 2014) was made to identify the 40 patients who underwent HTx with the intention of curing non-metastatic PCS.

The HTx group enlisted 6 patients FO and 40 patients FL who underwent HTx for PCS. The non-HTx group enlisted 7 patients with AS who received palliative treatments in our institution, their prognostic data were used to compare the results between palliative treatment and HTx for unresectable and non-metastatic AS. All the data from our institutional database and the literature were carefully studied and retrospectively reviewed.

In the HTx group, the overall survival time was either derived from the literature or calculated from the date of the HTx to the date of death or to the time of the report if the patient was still alive. In the non-HTx group, the overall survival time was calculated from the date of partial resection, biopsies or the diagnoses of local recurrences from previous R1 resections to the time of death. Considering the unresectable and non-metastatic tumor status of the non-HTx group was equal to the tumor status of the HTx group at the time of HTx, we set the survival time after the presentation of such tumor status in the non-HTx group as control in this study.

### Statistical analyses

The overall survival curves were calculated using standard Kaplan-Meier survival analysis. Univariate analysis using a log-rank test was performed on patients with available data. All the data were calculated using SPSS software (v20.0; IBM Corporation, Armonk, NY, USA).

## Results

In the HTx group, the clinical details of the 6 patients FO and the 40 patients FL [5–27] are outlined in Table 1 and the Additional file 1: Table S1 respectively. The mean age at HTx was 36 for 23 female patients and 19 male patients (data are not available for 4 FL [12, 26]). In the non-HTx group, the clinical details of the 7 patients are outlined in Table 2. The mean age at presentation is 49 for 4 female patients and 3 male patients.

**Table 1:** Treatment Courses of the 6 Patients Receiving HTx for primary cardiac sarcomas in Our Institution

Age/Sex	Histology (Grade)	Tumor Sites	Operations before HTX, Interval (mo)	Multimodal Therapies	Tumor Relapse (mo)	Outcome Survival (mo)
63/M	Synovial Sarcoma (G3)	LV, RV	Partial resection, 7	NT(pre-), XRT to metastasis	Lung (1)	D, 5
48/M	Angiosarcoma (G3)	RA, RV	Biopsy, 3	IAP(post-op)	Liver, Chest (4),	D, 5
27/F	Angiosarcoma (G3)	RA, RV	Biopsy, 2	IAP(post-op)	Lung (12)	D, 15
49/F	Undifferentiated Pleomorphic Sarcoma (G3)	RA, LA, LV	Partial resection, 5	Re-HTx at 36mo	PV (33), Liver, PV (40)	D, 43
49/F	Undifferentiated Pleomorphic Sarcoma (G2)	LA, LV	Partial resection, 9	No	-	D^a^, 18
61/M	Myxoid Liposarcoma, (G2)	RV	Partial resection, 3	No	-	Alive, 93

^a^Complicated by one episode of acute rejection, heart failure unrelated to tumor was the reason of death

*D* death, *F* female, *HT*, heart transplantation, *IAP* ifosfamide/doxorubicin/cisplatin, *LA* left atrium, *LV* left ventricular, *M* male, *NT* vinorelbine/cisplatin, *RA* right atrium, *RV* right ventricular, *XRT* radiation therapy

**Table 2:** Treatment Courses of the 7 Patients in Non-transplant Group

Age/Sex	Grade,	Status at Starting Point of Treatment	Chemotherapy	Radiation Therapy	Metastasis (mo)	Survival (mo)
40/F	G2	Recurrence at 6 mo after R1 resection	Yes	Yes	Bone (2)	D, 22
42/M	G3	Recurrence at 2 mo after R1 resection	Yes	Yes	Lung(14), bone (20)	D, 27
60/F	G3	Partial resection	Yes	Yes	Chest wall (2), brain (18)	D, 19
38/M	G3	Partial resection	No	No	-	D, 8
44/F	U	Partial resection	No	No	Brain (1)	D, 2
48/M	U	Biopsy	No	No	Lung (3)	D, 5
69/F	G3	Biopsy	No	No	-	D, 3

*D* death, *F* female, *M* male, *U* unkown

### Histology

In the HTx group, the histological subtypes presented in the literatures were re-reviewed and classified according to World Health Organization Classification System of Tumors of Soft Tissue proposed in 2013 [28], including angiosarcomas in 14 patients [6, 12, 13, 18, 19, 21, 23, 25, 26] (including 2 FO), undifferentiated/unclassifed sarcomas in 8 [5, 7, 8, 10, 14, 20] (including 2 FO), leiomyosarcomas in 5 [8, 13, 16, 17], fibroblastic/myofibroblastic tumors in 3 [9, 10, 22], adipocytic tumors [11] (including 1 FO), synovial sarcomas [24] (including 1 FO),rhabdomyosarcomas [12, 15] and intimal sarcomas [14] each reported in 2, osteosarcoma [16], malignant hemangiopericytoma [13] and neurofibrosarcoma [27] each reported in 1. The histological diagnoses of remaining 5 patients were unable to classify, including “myogenic sarcoma”[8], “myxosarcoma”[18], “sarcoma suggestive of muscle differentiation”[18], “histosarcoma”[16] and “intraventricular sarcoma”[16] which were described in the literatures.

In the HTx group, grading information was provided for 21 patients: 1 patient with grade 1 tumor [15], 5 patients with grade 2 tumor [8, 10] (including 2 FO) and 15 patients with grade 3 tumor [8, 10, 13, 14, 18, 22] (including 4 FO). The grading systems applied in this study included Federation Nationale des Centres de Lutte Contre le Cancer (FNCLCC) [8] (which was applied in our institution) and National Cancer Institute (NCI) criteria [10].

### Surgery

In the HTx group, all the patients undertook HTx. Because of infiltration of pulmonary vein or pulmonary artery, 3 patients undertook coinstantaneous lung transplantation [14] and 2 patients undertook coinstantaneous pneumonectomy [13]. In our institution (Table 1), R0 resections were confirmed by intraoperative frozen sections for the 4 patients with atrial sarcomas and by postoperative paraffin sections for the 2 patients with ventricular sarcomas. The information of margin status was provided in 9 patients FL, including R0 in 7 patients [5, 7, 12, 18, 23], R1 in 1 [18] and “positive” in 1 [10].

#### Immunosuppressive therapy

The information of the post-HTx immunosuppressive regents were provided in 23 patients [7, 9, 12, 13, 21–27] (including 6 FO). All patients received multidrug immunosuppressive therapy based on calcineurin inhibitor. Azathioprine or mycophenolate mofetil was used in combination with calcineurin inhibitor and prednisolone.

### Rejection episode

Seven patients [10, 13, 18, 22, 23] (including 1 FO) were reported to have one or more episodes of acute rejection after HTx.

### Chemotherapy and radiation therapy

In the HTx group, 18 patients [9, 12–14, 18, 19, 21, 23, 25, 26] (including 1 FO) received neoadjuvant chemotherapy and 11 patients [12, 13, 21, 23, 25, 26] (including 2 FO) received adjuvant chemotherapy. One patient [16] received pre-HTx or post-HTx chemotherapy. Two patients received pre-operational radiation therapy [15, 21]. Three patients [8, 14] (including 1 FO) received post-operational radiation therapy. In the non-HTx group, 3 patients received both chemotherapy and radiation therapy.

### Tumor recurrence

In the HTx group, 20 patients [7, 8, 12–14, 19, 23–25] (including 4 FO) experienced distant metastases. Four patients [16] developed metastases or recurrences. Data were unavailable for 2 patients [16, 18]. Twenty patients [5, 6, 8–13, 15, 17, 18, 20–22, 26, 27] (including 2 FO) were free of metastases at the time of the clinical endpoint or report, among them, 3 patients developed a local recurrence on the native residuals [12, 20] (including 1 FO). There were no secondary cancers reported at the time of the clinical endpoint or report.

### Post-HTx surgery for tumor relapse

Four patients received surgical resections of post-HTx distant metastasis [14, 23]. Two patients [12] (including 1 FO) received HTx for the second time for a local recurrence. The re-HTx FO received a partial resection with macroscopic residuals inside the pulmonary veins. The information of margin status was not provided for the other one [12].

### Survival

In the HTx group (*n* = 46), parametric estimate of overall survival was as follows: 61 % ± 7 % at 1 years; 44 % ± 8 % at 2 years; 26 % ± 8 % at 5 years. The overall median survival was 16 months (range 2–112 months). Among it, the median survival for the 6 patients FO was 15 months (range 5–93 months), which was not different from the median survival of 16 months (range 2–112 months) for the 40 patients FL (*P* = 0.768).

In the HTx group, the 30-day or hospital mortality after HTx was zero. Twenty-nine (63 %) patients [7, 8, 12–14, 16, 18–20, 23–25] (including 5 FO) had died by the time of the report. The cause of death involved tumor local recurrences or distant metastases for 24 patients, unknown reason [16, 18] and heart failure unrelated with tumors [13] (1 FO) respectively for 2 patients, pneumonitis for 1 patient [14]. The remaining 17 (37 %) patients [5, 6, 8–11, 15, 17, 18, 21, 22, 26, 27] (including 1 FO)were still alive at the time of the report, the median follow-up time was 20 months (range 2–112 months). In the non-HTx group, all the 7 patients died of tumor progressions.

**Fig. 1:**
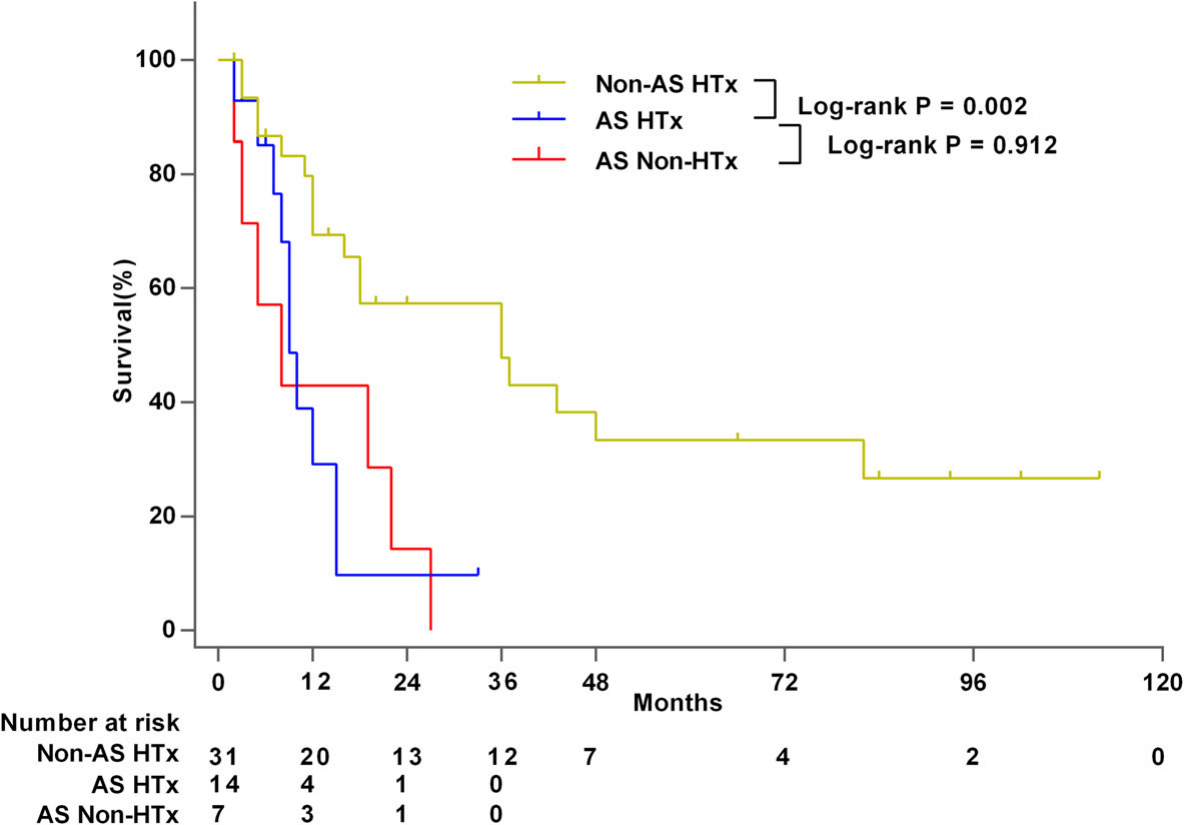
Overall survival after HTx for AS compared with HTx for non-AS primary cardiac sarcoma and palliative therapy for AS. *HTx* heart transplantation, *AS* primary cardiac angiosarcoma

The median survival after HTx for AS(*n* = 14) was 9 months (range 2–33months), which was less than the 36 months (range 2–112months) for non-angiosarcoma PCS (*n* = 31) (*P* = 0.002) (Fig. 1). And it was not much different from the median survival of 8 months (range 2–27months) in the non-HTx group(*n* = 7) (*P* = 0.912) (Fig. 1).

**Fig. 2:**
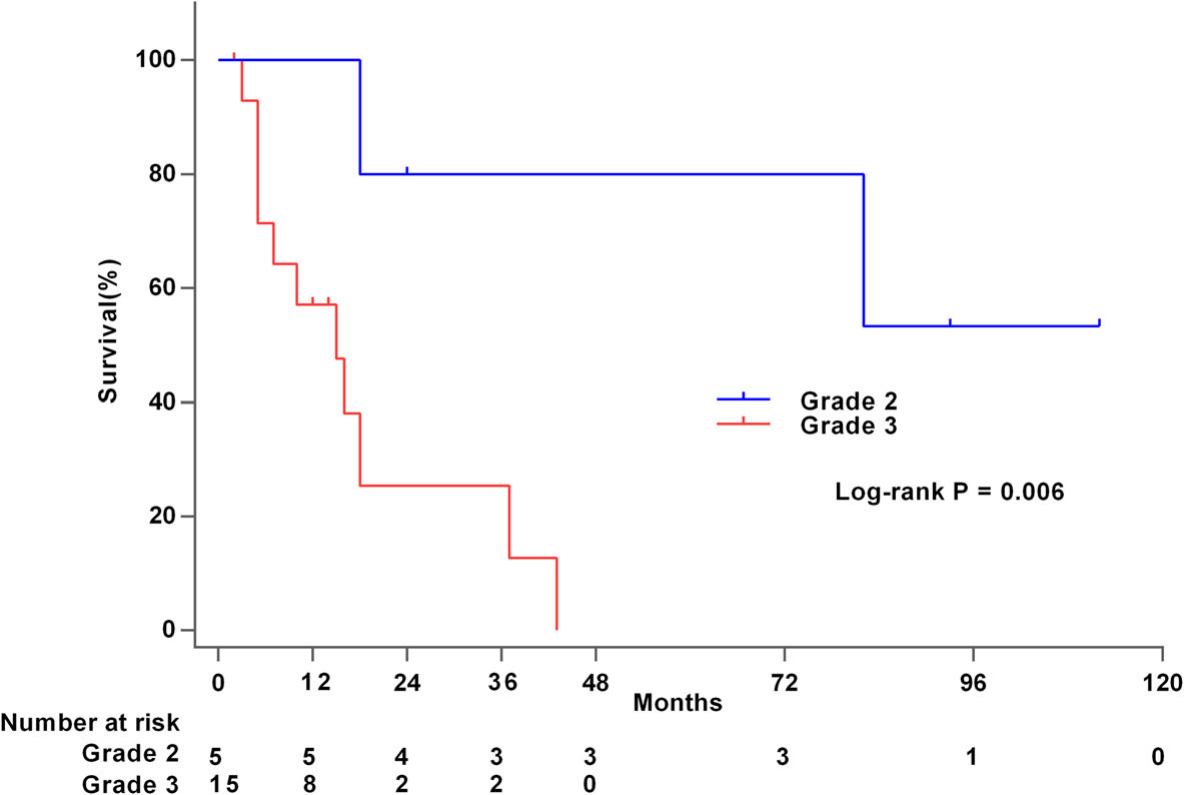
Overall survival after HTx for grade 2 primary cardiac sarcoma compared with HTx for grade 3. *HTx* heart transplantation

The mean survival of grade 2 PCS (*n* = 5) who received HTx was 85 months (range 18–112 months), which was much longer than the 18 months (range 2–43 months) for grade 3 PCS (*n* = 15) (*P* = 0.006) (Fig. 2).

In the HTx group, one patient with uncertain data [16] was excluded from the analysis, neoadjuvant and adjuvant chemotherapy presented in this study failed to display their survival difference after HTx. The influence of chemotherapy was analyzed separately on patients with histologic types other than AS and grade 3 tumors. No survival differences were found in these two groups as showed in the whole cohort (Table 3).

**Table 3:** Comparison of survivals in different groups who received heart transplantation for primary cardiac sarcoma with or without chemotherapy

Patients	Survival (mo) median (range), (cases)		Log-rank *P* value
*Neoadjuvant chemotherapy*	*Yes*	*No*	
All	15 (2–84), (*n*–18)	18 (2–112), (*n* = 27)	0.210
Non-angiosarcoma	36 (5–84), (*n* = 8)	43 (2–112), (*n* = 22)	0.462
Grade3	7 (5–37), (*n* = 6)	15 (2–43), (*n* = 9)	0.731
*Adjuvent chemotherapy*	*Yes*	*No*	
All	15 (5–36), (*n* = 11)	36 (2–112), (*n* = 34)	0.088
Non-angiosarcoma	18 (18–36), (*n* = 2)	37 (2–112), (*n* = 28)	0.407
Grade3	10 (5–18), (*n* = 5)	16 (2–43), (*n* = 10)	0.169

Values are presented as median (range)

## Discussion

HTx is an uncommon treatment for PCS which are also rare disease. Only 40 cases of HTx for non-metastatic PCS were reported in the literatures up to date. Previous studies were based on data of single case or small case series [5–27]. Our study offers a series of patients who undertook HTx for PCS in our institution and those presented in the literature. Although deriving survival time from previously reported retrospective case series is questionable on reliability, the combined data in this study provide a chance to perform a first-ever survival analysis of HTx for PCS.

Angiosarcoma accounts for nearly half of PCS [1] and has a very poor prognosis [29]. The fast local infiltration and early metastases attribute to the poor result of current therapies. For non-metastatic AS, the median survival after non-HTx treatments was 19.5 months according to Nicole et al. [29]. The median survival of HTx for the 14 cases of AS in the present study was merely 9 months. That was much less than that of HTx for other histologic types of PCS and not much different from the unresectable AS who received palliative therapy in our institution. According to previous reports [2, 3, 29], non-metastatic AS who received R0 resections had much better survivals than those who received THx in the present study. The reason why HTx was chosen is that it has the capability of R0 resection for the unresectable tumor. But the disappointing result of HTx for AS does not reach even close to the original expectation.

Among the 14 patients who received HTx for AS, 10 patients developed distant metastases within 1 year after HTx [12, 13, 19, 23, 25]. Three patients were reported free of metastases by the time of reports [6, 18, 26] with relatively short follow-ups from 3 to 8 months. The only one successful result [21] of 33 months free of disease after HTx couldn’t be repeated. Early metastasis is the major reason for poor prognosis of HTx for AS. It seems that the influence of immunosuppressant agents on tumor spreading [30] is substantial for this histologic type of PCS. The poor result of the HTx for AS group failed to change for the better by means of neoadjuvant or adjuvant chemotherapy which was undertaken in 13 out of the 14 patients. Base on the current available data, patients with AS seem unsuited for HTx due to the high propensity to develop metastases.

Incomplete resection followed by multimodality treatments such as radiation or chemotherapy can achieve reasonable long-term survivals for AS with regional extension, which is evidenced by the 3 cases whose survivals ranged from 19 to 27 months in the non-HTx group and a 4-case series in the previous report [31] who survived up to 25 to 81 months (average, 51 months). These facts imply that a better multimodal strategy is needed rather than HTx for unresectable AS.

Radical resections of cardiac sarcomas with regional extension can achieve R0 resections and have shown good outcomes [3, 32]. The entire right atria and up to 30 % of right ventricle can be removed; surrounding structures such as the cardiac valve, coronary artery and great vessel can be resected. Cardiac autotransplantation allows for excellent exposition and confident excision of left atrial sarcomas, which seems impossible through conventional approaches. Although it is a technical challenge, autotransplantation can provide better prognosis than HTx because of the avoidance of immunosuppressive agents, especially for the aggressive histologic types such as angiosarcoma whose metastatic rate will be greatly aggravated by immunosuppression. Such strategies can spare some potential candidates in who the tumor is restricted within the left atrium from HTx.

PCS are such lethal diseases with a median overall survival of only 6 months in patients with diagnoses of PCS registered in the Surveillance, Epidemiology and End Results database [1]. Complete resections gained just 24–36 months of median survivals [2–4, 33]. The current study showed a median survival of 36 months after HTx in patients with non-AS PCS. Furthermore, 42 % (13/31) of the patients with non-AS PCS were still alive at the time of the report, these patients might be the longer survivors and increase median survival after complete follow-up. Therefore, for histologic type other than AS, the role of HTx remains undefined and current results deserve further investigation.

There is a small group of soft tissue sarcoma subtypes which are less aggressive with local growth patterns, weaker tendency to metastasize than other subtypes. HTx for these types of PCS is of great benefit. For instance, survival and metastases rates of myxofibrosarcoma were 77 and 15 % respectively at 5 years after surgery at extracardiac sites [34]. The patient with cardiac myxofibrosarcoma receiving HTx in the literature [10] survived at least 112 months. Resections of myxoid liposarcoma at extracardiac sites [35] have 91 % of 5-year survival rate and 77 % of 10-year metastatic-free rate. The patient of myxoid liposarcoma from our institution has survived up to 93 months after HTx and she is still alive up to the date when this article is being written.

Sarcomas grading provides more information for predicting the prognosis in addition to histologic typing [28]. In the cases presented in this study, HTx candidates for PCS were strictly selected for no distant metastases, but metastasis other than local recurrence is the major detriment of the prognosis after HTx. Grading of soft tissue sarcoma is helpful to predict the probability of distant metastases [28]. In the largest survey of PCS receiving non-HTx therapies [1], the tumor grade (poorly differentiated) was found to be an independent prognostic factor. In the current study, patients of grade 2 PCS after HTx are learnt to survive much longer than those of grade 3 PCS. Although there are some differences among different grading systems such as FNCLCC and NCI applied in this study, the result of the current study implies that grading is a predictor of prognosis and helpful to select the patients who stand a chance to profit more from HTx. It is worth mentioning that the only patient of grade 1 PCS receiving HTx survived at least 102 months [15]. Since precise histologic diagnoses of PCS are often obtained by biopsy or incomplete resection in the previous stage, it provides a good chance to prudently select the patients with PCS who will benefit from HTx.

The role of neoadjuvant or adjuvant chemotherapy for soft tissue sarcomas remains controversial. Aiming at the aggressive nature of PCS, the use of neoadjuvant chemotherapy is supposed to shrink the tumor, increase resectability and neutralize micrometastases. Undesirably, it failed to show any direct proof of survival improvement [2, 3]. The analyses in the current study show no benefit from neoadjuvant chemotherapy in the case of HTx. In order to eliminate the statistical anomaly caused by low grades which are less aggressive and may profit more from HTx rather than from chemotherapy, or by AS whose poor prognosis is determined by intrinsic high malignancy, the relevant analyses have been performed on patients with histologic types other than AS and grade 3 tumors, and the results are equivalently ineffective. Meanwhile, the analysis of adjuvant chemotherapy has produced the same result of futility. Hope lies in the new chemo agents and target therapies designed for histologic subtypes.

Post-HTx local recurrences were less frequent compared to distant metastases in the present study, which might be attributed to the R0 capability of HTx or the relative short-term follow-ups. The recurrences are usually located at the native side of the anastomosis, such as the residuals of the pulmonary veins. Re-HTx which still anastomoses the pulmonary veins to the left atrium cannot guarantee a border free of disease. Although the two cases of re-HTx (1 FL [12] and 1 FO) were free of local occurrence 28 months and 33 months after the first HTx seperately, local recurrences of the second time occurred several months after re-HTx. Repeated lung and heart transplantation might be better in term of the resection range.

According to the current ISHLT (The International Society for Heart & Lung Transplantation) data [36], the median survival after HTx was 11 years for all and 13 years for those surviving the first year, which are much better than the mean survival of 7 years for the grade 2 PCS in the present study. It means that even the prognosis of the histologic types which benefit the most from HTx are much poorer than the prognosis of the usual HTx recipient. In the context of donor shortage, it raises doubts to perform HTx for recipients with PCS instead of recipients with non-tumor causes who gain the maximum benefit. For the “favourable” histologic types of PCS, HTx with a marginal donor hearts may be a more acceptable strategy.

The current study has inherent limitations. Although this study has presented 6 patients from our institution and almost all the cases presented in the literatures with the data pooled from them to the greatest extent, it is a small number retrospective study. Analysis of combined data from heterogenous patients described in the literature and data from our institution was inevitably unreliable. However, the combined data have provided us a chance to perform a survival analysis of this unusual treatment for these rare diseases, otherwise it is difficult to perform such analysis with a small amount of data from a single institution. The analyses in this study can only be exploratory and serve to provide more information regarding HTx strategy for patients with these fatal PCS.

## Conclusions

The researches so far have indicated that HTx for PCS harbors a relatively wide spectrum of prognosis. Our findings suggest that AS may be not a proper indication of HTx though it is the most common histologic type of PCS. The role of HTx for histologic types other than AS remains undefined. Lower grade and less aggressive histologic types benefit more from heart transplantation. Current neoadjuvant or adjuvant chemotherapy agents may fail to improve prognosis after HTx.

## Additional file

Additional file 1: Table S1. Patients undergoing HTx for PCS Presented in the literatures. (DOCX 19 kb)

## Abbreviations

AS: Primary cardiac angiosarcoma
FL: From the literature
FNCLCC: Federation Nationale des Centres de Lutte Contre le Cancer
FO: From our institution
HTx: Heart transplantation
NCI: National Cancer Institute
PCS: Primary cardiac sarcomas.
