# Supplementary material for: Survival after heart transplantation for non-metastatic primary cardiac sarcoma
Source: J Cardiothorac Surg. 2016 Oct 3;11:145. doi: 10.1186/s13019-016-0540-x (PMC5048623; doi:10.1186/s13019-016-0540-x)
Supplement: Additional file 1: Table S1. — Patients undergoing HTx for PCS Presented in the literatures. (DOCX 19 kb) [file 13019_2016_540_MOESM1_ESM.docx]

Supplemental Table Patients undergoing HTx for PCS Presented in the literatures

| Literature | Age(y)/Sex | Histology(Grade) | Adjuvant Chemotherapy | Tumor  Relapse (mo) | Outcome, Survival (mo) |
| --- | --- | --- | --- | --- | --- |
| Lok *et al*.[5] | 19/F | Malignant Mesenchymoma, NOS | No | No | A, 48 |
| Agaimy *et al*.[6] | 36/F | Angiosarcoma | No | No | A, 3 |
| Lee *et al*.[7] | 42/M | Malignant Fibrous Histiocytoma | No | Multiple Mets (8) | D, 11 |
| Winther *et al*.[8] | 34/F | Myogenic Sarcoma (G2) | No^a^ | Brain (72) | D, 82 |
|  | 56/F | Leiomyosarcoma (G2) | No | Bone (NS) | A, 24 |
|  | 55/F | Pleomorphic Sarcoma, NOS (G3) | No | No | A, 14 |
| Coelho *et al*.[9] | 25/F | Fibrosarcoma | Yes(pre) | No | A, 84 |
| Zhang *et al*.[10] | 6/F | Myxofibrosarcoma (G2) | No | No | A, 112 |
|  | 28/F | Sarcoma, NOS (G3) | No | No | A, 2 |
| Hoffmeier *et al*.[11] | 8/M | Liposarcoma | No | No | A, 6 |
| Mazuecos *et al*.[12] | NS | Rhabdomyosarcoma^b^ | Yes(pre- and post-op) | Native Atrium (28)  (7 after re-HTx) | D, 36 |
|  | NS | Angiosarcoma | Yes(pre- and post-op) | Brain (NS) | D, 12 |
|  | NS | Angiosarcoma | Yes(pre-) | Brain(NS) | D, 9 |
| Uberfuhr *et al*.[13] | 28/F | Angiosarcoma (G3) | Yes(post-op) | Mets (NS) | D, 10 |
|  | 42/F | Malignant Hemangiopericytoma (G3)^c^ | Yes(post-op) | Mets (NS) | D, 18 |
|  | 44/M | Angiosarcoma (G3) | Yes(pre- and post-op) | Mets (NS) | D, 7 |
|  | 35/M | Leiomyosarcoma (G3) | Yes(pre-) | No | D^d^, 37 |
| Talbot *et al*.[14] | 40/M | Intimal Sarcoma^e^ | Yes(pre-) | Abodomen (36) | D, 48 |
|  | 37/F | Spindle Sarcoma (G3) ^e^ | Yes(pre-) | Lung (6) | D,16 |
|  | 45/M | Intimal Sarcoma (G3) ^e^ | Yes(pre-) | Brain (1.5) | D^f^, 5 |
| Grandmougin *et al*.[15] | 33/M | Rhabdomyosarcoma (G1) | No | No | A, 102 |
| Gowdamarajan *et al*.  [16] | 64/M | Intraventricular Sarcoma | No | Unkown | D^g^, 3 |
|  | 7.5/M | Leiomyosarcoma | Yes(pre- or post-op) | Mets or local relapse | D, 11.5 |
|  | 28/F | Osteosarcoma | No | Mets or local relapse | D, 11.5 |
|  | 9/M | Leiomyosarcoma | No | Mets or local relapse | D, 11.5 |
|  | 61/F | Histosarcoma | No | Mets or local relapse | D, 36 |
| Babatasi *et al*.[17] | 47/M | Leiomyosarcoma | No | No | A, 20 |
| Michler *et al*.[18] | 42/F | Myxosarcoma (G3) | Yes(pre-) | No | A, 16 |
|  | 64/M | Sarcoma of Muscle Differentiation (G3) | No | Unkown | D^g^, 3 |
|  | 41/F | Angiosarcoma | Yes(pre-) | No | A, 6 |
| ALmenar *et al*.[19] | 29/F | Angiosarcoma | Yes(pre-) | Lung (1) | D,2 |
| Bachet *et al*.[20] | 35/M | Malignant Histocytofibroma | No^a^ | Native IVC (4) | D, 8 |
| Baay *et al*.[21] | 34/M | Angiosarcoma | Yes(pre- and post-op) | No | A, 33 |
| Aufiero *et al*.[22] | 31/F | Fibrosarcoma (G3) | No | No | A, 12 |
| Crespo *et al*.[23] | 31/M | Angiosarcoma | Yes(pre) | Brain (5) | D, 8 |
|  | 32/M | Angiosarcoma | Yes(pre- and post-op) | Brain (8) | D, 9 |
| Siebenmann *et al*.[24] | 31/F | Synovial Sarcoma | No | Chest (2) | D, 2 |
| Armitage *et al*.[26] | 13/NS | Angiosarcoma | Yes(pre- and post-op) | No | A,8 |
| Horn *et al*.[25] | 12/M | Angiosarcoma | Yes(pre- and post-op) | Mets (< 12) | D, 15 |
| Aravot *et al*.[27] | 43/F | Neurofibrosarcoma | No | No | A, 66 |

^a^ Chemotherapy and tumor resection were undertaken as initial treatments; ^b^ A secondary HTx was performed at 28 months for recurrence; ^c^ HTx and left pneumonectomy combined; ^d^ This patient received HTx with right pneumonectomy but died of right heart failure afterwards; ^e^ heart and lung transplantation combined; ^f^ died of pneumonitis; ^g^ unknown causes.

*A* alive at the time of report, *D* died of tumor progression, *IVC* inferior vena cava, *Mets* distant metastases, *NOS* not otherwise specified, *NS* not specified in the report.
